# Malleability of the self: electrophysiological correlates of the enfacement illusion

**DOI:** 10.1038/s41598-018-38213-y

**Published:** 2019-02-08

**Authors:** Ilaria Bufalari, Anna Laura Sforza, Francesco Di Russo, Lucia Mannetti, Salvatore Maria Aglioti

**Affiliations:** 1 Dipartimento dei Processi di Sviluppo e Socializzazione, Università degli studi di Roma “La Sapienza”, Rome, Italy; 2 IRCCS Fondazione Santa Lucia, Rome, Italy; 3 Dipartimento di Scienze Motorie, Umane e della Salute, Università degli Studi di Roma “Foro Italico”, Rome, Italy; 4 Dipartimento di Psicologia, Università degli studi di Roma “La Sapienza”, Rome, Italy

## Abstract

Self-face representation is fundamentally important for self-identity and self-consciousness. Given its role in preserving identity over time, self-face processing is considered as a robust and stable process. Yet, recent studies indicate that simple psychophysics manipulations may change how we process our own face. Specifically, experiencing tactile facial stimulation while seeing similar synchronous stimuli delivered to the face of another individual seen as in a mirror, induces ‘enfacement’ illusion, i.e. the subjective experience of ownership of the other’s face and a bias in attributing to the self, facial features of the other person. Here we recorded visual Event-Related Potentials elicited by the presentation of self, other and morphed faces during a self-other discrimination task performed immediately after participants received synchronous and control asynchronous Interpersonal Multisensory Stimulation (IMS). We found that self-face presentation after synchronous as compared to asynchronous stimulation significantly reduced the late positive potential (LPP; 450–750 ms), a reliable electrophysiological marker of self-identification processes. Additionally, enfacement cancelled out the differences in LPP amplitudes produced by self- and other-face during the control condition. These findings represent the first direct neurophysiological evidence that enfacement may affect self-face processing and pave the way to novel paradigms for exploring defective self-representation and self-other interactions.

## Introduction

A coherent visual representation of one’s own face is formed and continuously updated by matching facial somatosensory and motor experiences with what is seen in the mirror^1^. Ultimately, this process allows self-face mirror recognition, an ability that index self-awareness of one’s own physical appearance^2^ and that seems to precede more complex forms of self-consciousness and social behavior^3^.

Behavioral studies using reaction times indicate that the self-face is recognized faster^4^ and grabs and retains attention longer than even highly familiar other faces^5,6^; is processed in a distinct right-dominant but largely bilaterally distributed occipito-temporo-parietal-frontal circuit^7,8^; and is misrecognized rarely and almost exclusively in patients with severe neurological^9^ or psychiatric disorders^10^.

Although self-face recognition is considered a rather stable process, recent studies show that deriving the self from one’s own face may be an inherently plastic process^11^. For example, experiencing tactile facial stimulation while seeing similar synchronous stimuli on the face of another individual (Interpersonal Multisensory Stimulation; IMS) induces ‘Enfacement’, an illusion where people report the subjective experience of ownership of the other’s face and exhibit a bias in attributing to the self facial features of the other^12–19^. This effect would occur because the congruency between observed and felt touch generates surprise. Since participants cannot move to prove themselves the observed face is not theirs, their brain may attempt to minimize surprise by changing self-face representation to include the other person’s facial features^20^.

Only two studies have so far investigated the neural correlates of the Enfacement illusion via synchronous visuo-tactile^21^ and visuo-motor^22^ IMS. Apps and colleagues provided important evidence on the neural correlates of enfacement, although they recorded BOLD activity exclusively during facial IMS^21^. Importantly, however, only investigating the neurocognitive processes associated with the self-face after the illusion has been induced may provide direct evidence for enfacement-induced changes of visual self-face representation.

Given its excellent time resolution, the event related potential (ERP) technique is ideal to explore the different stages of face processing. Interpreting face related ERPs components within classical^23^ and revisited face recognition models^24,25^, is not easy because of the seeming contradiction between different studies that use different experimental paradigms and tasks (participants may adopt different strategies depending on which dimension of the face is relevant to the task; for example, asking to identify if the observed face is familiar may not require the same processes necessary to identify whom the face belongs to). Different studies suggest that: (i) early activity (as indexed by P100 and N170 components) may reflect an initial featural and configural encoding stage^26–28^; (ii) activity in the 200–500 ms latency range (as indexed by P/N250 and N400f components) seems to reflect a subsequent matching stage, where the constructed representation is compared to stored structural representations and a familiar face is recognized^29,30^; (iii) long latency range components such as N400, P300 and LPP^29,31,32^ seem to reflect the activation of a cognitive route that provides access to semantic and biographical information about the face (i.e., to identity nodes) and of a second separate route – interconnected with the first one - responsible for the generation of an affective response toward the familiar face. In fact, the late processing stages - particularly P300 and LPP ERPs- have been related to a variety of functions: from faces’ attractiveness discrimination^33–35^, to emotional, affective evaluation^36,37^ and detection of faces’ motivational relevance^37,38^, to dynamic allocation of attention^39,40^ and memory encoding^41^.

Interestingly when comparing the self with other familiar or unknown faces^42–49^ the visual self-face processing differs from other face processing at different levels, even though no univocal link between specific ERPs components and the different perceptual and cognitive processes involved in face recognition was established. Indeed, the studies that compared early and late ERPs evoked by the self, familiar (and/or famous) and unknown faces^42–49^ showed that the self-face differs from all other faces (i.e., personally known/experimentally acquired and unknown faces; i.e., *self-specificity effect*), and that the self and familiar faces are distinguished from unknown faces (i.e., *familiarity effect*) within the first 200–250 ms from the presentation of the face stimuli. However, the occurrence of these effects in time varied across studies. Some studies found self-specificity already at the level of the N170^43,45^ or of the N250 (first half of the experimental trials^30^). Other studies found familiarity effects over the N170^42^, the N250 (second half of the trials^30^) and delta event-related synchronization (which is interpreted to reflect N250 activity, in 200–800 time window^50^). It is worth noting that the above-mentioned studies employed a variety of control stimuli [from personally known/relevant familiar faces, to personally unknown but still familiar (e.g. famous) or completely stranger faces] and experimental tasks [e.g., from passive observation^42,51^, to familiarity detection task^28,43,46,52^, to traditional oddball task with the self being the target or non-target face^44,48,50,53^, to identification of face orientation^54–56^ or emotional expression^28,45^]. Such a large variety of conditions may lead to different strategies depending on which dimension of the face is relevant for the task at stake and may be at the basis of the variability of familiarity or self-specificity effects. Importantly, however, these studies converged in finding that the distinction between self, familiar (and/or famous) and unknown faces^42–49^ (i.e., the *face identification effect*) occurs always after the self-specificity and familiarity effects and in the long latency range (350–800 ms) comprising slow and widespread long-latency components, such as the LPP, P3, and N400 components depending on the experimental paradigm^44,45,47^ [but see^42,43^ for face identification effects in earlier time range]. Also, the above mentioned studies converged to show that the face identification effect is always reflected in maximal ERPs amplitudes for the self-face with gradually and significantly decreasing amplitudes for decreasing levels of familiarity of the other faces. The studies that analyzed the self with respect to only familiar^55^, famous^51^, or target family faces (mother and father^48^), or that analyzed only the late P300 ERP component^46,53^ also found highest amplitudes for the self-face with respect to other faces in the late time windows (P300 and LPP). It has been proposed that P300 may index prioritized attentional resource allocation to self-relevant – not only facial - information (e.g., vocalized name^46,57,58^ or written autobiographical information^59^). The LPP, instead, being modulated by the intensity of emotional content of the stimuli^60^, may reflect a global inhibition of potentially competing representations which allows more selective processing of the emotional stimulus that evoked it^61^. Importantly, the only study that used a face identification task to compare the self with an equally matched face in terms of personal relevance, emotional salience, life-time exposure, age and gender (i.e., the co-twin face) and that analyzed several ERPs components^47^, found that the self-face differed from the co-twin face only at long latency range (400–600 ms). Importantly, the late electroencephalographic activity (400–600 ms) not only differentiated the self from a dizygotic co-twin’s face but also the current from the past self face. In contrast, such activity did not differentiate the current from the past co-twin’s face^47^. Taken together, these results may unravel another important process indexed by the late slow components, e.g. the process of identifying the self-face, by matching the observed self-face with its online and offline representations. In sum, the effects found in the late latency range have been associated to the memory retrieval of self-face specific information^30,44–46,48,50,53,54,62^, as well as to allocation of attention to self-relevant – not only facial – information^46,57–59^. Thus, late ERPs activity suggests that processing self- vs. other-related cues implies different forms of storage and different processing of relevance-related information.

In a recent study that used ERPs, Serino and colleagues (2015) investigated whether experiencing agency over an unfamiliar avatar’s face (i.e. a modified version of the original enfacement effect) may change the early electrophysiological processing of self-avatar morphed faces, in the time range where previous literature found self-specificity and familiarity effects. It is worth noting that given the type of experimental paradigm adopted in this study, only early stages of face processing were investigated. More specifically, faces were presented very shortly (for 200 ms) and a two-alternative forced choice response (“self”/“avatar”) was requested in the next 600 ms. Thus, ERPs later than 200 ms were not visible or were contaminated by stimulus offset and by participants’ response preparation and execution. Interestingly, the authors found that configural processing of 50% self- 50% other morphed faces changed between synchronous and asynchronous condition, as indexed by modulations of the N170, a component arising from right inferotemporal-occipital cortex in the region of the fusiform gyrus^22^. In particular, they found that N170 was heightened in amplitude and anticipated when images morphing the self and the avatar’s faces were shown after experiencing synchronous vs. asynchronous movement with the avatar’s face. Such effect was found to be linked to activation of the sensory-motor cortex during the synchronous movement condition. No effect of illusory agency was found, instead, over P100 or P/N250 components.

Despite the above mentioned significant difference, the question of whether morphed face processing became similar to the self-face processing as a result of experiencing illusory agency over the avatar’s face remains unanswered. Indeed, in Serino and colleagues’ study^22^ such comparison was not reported and, contrary to previous findings^28,42,43,45^, the N170 showed generally higher amplitudes for the unknown (avatar) face than for the self-face. Furthermore, both morphed images judged as belonging to the self and to the avatar contributed to the “morphed face” category. Thus, the critical process of illusory attribution to the self of images containing 50% of the avatar’s face might have not been disentangled. Indeed, comparing morphed faces on the bases of perceptual judgments would have revealed whether morphed images attributed to the self were processed differently from morphed images attributed to the avatar, and similarly to self-images.

In the present study, we were specifically interested in testing whether experiencing Interpersonal Multisensory visuo-tactile Stimulation may change late stages of neurocognitive processing which previous literature specifically associated with processing of the self: not only with general self-related and self-relevant information^46,57–59^ but importantly also with self-face identification (i.e., the *face identification effect*)^30,44–46,48,50,53,54,62^ and with the process of identifying the self-face by matching the observed self-face with its online and offline representations^47^.

To explore this issue, we recorded visual event-related potentials (ERPs) evoked by the presentation of the participant’s own face and the face of a same gender, personally known individual during a subtle self-other face discrimination task performed immediately after synchronous and asynchronous IMS was delivered. Participants observed images from the self-other face morphing continuum for 1000 ms, i.e., an amount of time which consented to record not only early but also late ERPs, and after a delay of 1000 ms they had to report (using a Visual Analogue Scale, VAS) the exact amount of self and other facial features contained in each image. Thus, we chose a task that prompted participants to perform a fine-grained analysis of the observed face stimulus and to compute the overall distance between observed face and internal self-face representations to provide an accurate and final response. Even though we were specifically interested in testing the effect of IMS on components occurring in the late temporal window where self-identification seems to occur, we also wanted to exclude that any further IMS-related effect occurred in earlier time windows where self-specific, familiarity, as well as illusory facial agency^22^ effects were found.

We hypothesized that synchronous IMS updates the self-face representation to include other’s facial features, a process that would be indexed by modulation of ERPs in the long-latency windows where self-face identification and the matching of observed self-face with offline and online self-face representations seem to occur. In particular, we expected that: (i) in line with previous literature, in the control asynchronous condition the late ERPs would be highest for the self and progressively lower for the friend and the morphed face; and (ii) synchronous with respect to asynchronous IMS would reduce the amplitudes evoked by self-face which may become similar to those evoked by the friend face.

## Method

### Participants

Twenty naïve, normal or corrected-sighted, right-handed^63^ volunteers participated in the experiment [10 same-sex pairs, 10 females; (mean age ± st.dev.) (27.21 ± 3.99)]. Sample size was estimated on the basis of previous behavioral studies investigating the effect of a/synchronous facial IMS over self-other face recognition/discrimination abilities^12,15,17,64–66^ and of electroencephalographic studies investigating the processing of self, other and familiar faces^30,42–45,47,49–51,53–56,62,67,68^. Participants signed an informed consent for study participation and were paid 10 *euro* per hour. The procedures were approved by the ethics committee of Santa Lucia Foundation (Rome) and were in accordance with the ethical standards of the 1964 Declaration of Helsinki. To avoid that greater familiarity with one’s own vs. stranger’s faces could affect *per-se* self-other face discrimination abilities, we recruited pairs of participants who were familiar to each other (please see Supplementary Table S1).

### Experimental Design

**Figure 1:**
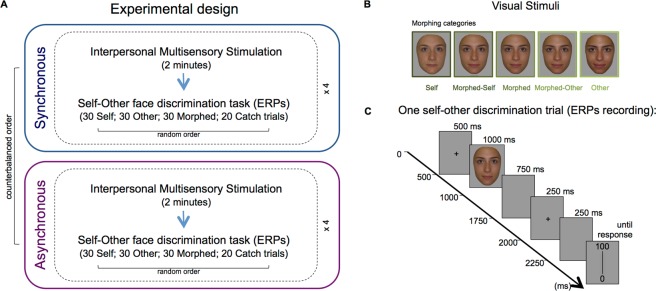
(**A**) Experimental Design. Participants were seated in front of each other. A trained experimenter standing between the two, touched their cheeks with two wooden sticks either synchronously (illusion condition) or asynchronously (no illusion condition). The synchronous and asynchronous Interpersonal Multisensory Stimulation (IMS) conditions were administered in separated runs and in counterbalanced order across participants. Each a/synchronous run consisted of four blocks (dashed rectangles). Each block comprised 2 minutes of IMS stimulation immediately followed by the self-other discrimination task of 110 faces [30 images for Self, Other, and Morphed face intervals (for a total of 90 images) plus 10 images from each of the two categories adjacent to the critical Morphed face interval (catch trials)] presented in random order. Participants had to evaluate along a 0–100 VAS the amount of self or other (depending on group) facial features contained in each face and ERPs were recorded from the visual presentation of faces. (**B**) Examples of visual stimuli. Five relevant categories from the original morphing continuum were selected: 1) the Self Face; 2) the Other face; 3) the Morphed face (on average 45% of Self); and as catch trials 4) the Morphed-Self face (on average 55% of Self); and 5) the Morphed-Other face (on average 35% of Self). (**C**) The timeline of a typical self-other face discrimination trial.

A schematic representation of the experimental design is given in Fig. 1A and details are provided in the following paragraphs. Electrodes’ placement and preparation were arranged at the beginning of the experimental session and before any IMS stimulation and self-other discrimination tasks. Synchronous and asynchronous IMS were administered in separated runs. Each run comprised four blocks of the same type of IMS. Runs’ order was counterbalanced across participants. Each block comprised 2 minutes of a/synchronous visuo-tactile IMS immediately followed by the self-other face discrimination task. ERPs were recorded during the self-other discrimination task and evoked by the visual presentation of the face images.

#### Interpersonal multisensory visuo-tactile stimulation

Each participant was seated in front of a table and faced his or her partner from a distance of about 140 cm. An experimenter stood between the two participants and repeatedly touched the subjects’ cheeks with two wooden sticks either synchronously (i.e., with a mirror correspondence between the touch on the two faces; illusion condition) or asynchronously (about 1 second delay; no illusion condition). As in previous studies, tactile stimuli were delivered manually by an experimenter who had undergone metronome training to deliver 1 second strokes, to synchronize the strokes between two faces, and to deliver them with 1 second of asynchronicity. Subjects were instructed to fixate on their partner’s face and concentrate over the observed tactile stimulation while wearing a rigid white-paper funnel around the eyes, which prevented the lateral view of the touches on their faces.

#### Self-Other discrimination task

In line with our previous behavioral study, a self-other face discrimination task particularly appropriate to investigate subtle changes in self-other face discrimination ability was employed immediately after each a/synchronous stimulation block^14^. In our previous study^14^, we found that participants reported higher amount of self facial features in the morphed faces containing 55% Other-45% Self after synchronous with respect to asynchronous and no-stroking conditions, where they could report the exact amount of self and other features. The synchronous-IMS specifically affected the 55% Other-45% Self face category, but no other morphed faces or the 100% Self and 100% Other faces.

In the present study, visual ERPs were recorded during the behavioral task (i.e., ERPs were evoked by the presentation of faces taken from the self-other morphing continuum) immediately after the synchronous and asynchronous IMS stimulation. Since we needed the repetition of many stimuli for each condition to collect clear ERPs, to keep the experiment as short as possible we decided to have as control condition only the asynchronous one considering that the self-attribution scores did not differ between asynchronous and baseline conditions in our previous behavioural study^14^.

Visual stimuli were tailored individually for each pair of participants. Frontal pictures of participants’ faces with a neutral expression were taken with a digital camera in the same environment with same lighting conditions and were edited using Adobe Photoshop CS to remove external features (hair, ears) and create a uniform grey background. For each pair of participants, both the self-face and friend’s face were scaled to equalize pupil-to-pupil distance and to ensure that the vertical midline of the image bisected the face. The scaling was applied to the whole face in order to maintain the original proportion. Then, for each pair different degrees of digital morphing between the two picture faces were created using Abrosoft Fantamorph 4.0. Five relevant categories from the original morphing continuum were selected (Fig. 1B): (1) the Self Face (participant’s own face: from 100% to 91% of the Self-face; on average 95% of Self); (2) the Other face (the pair participant face: from 9% to 0% of the Self-face; on average 5% of Self); the Morphed face (faces from the mixed ambiguous region where the *enfacement* effect was originally found; from 49 to 40% of the Self-face, on average 45% of Self^14^); (4) the Morphed-Self face (from 60% to 51% of the Self-face; on average 55% of Self); and (5) the Morphed-Other face (from 39% to 30% of Self; on average 35% of Self). Faces belonging to the last two categories, which are adjacent to the Morphed face category but where no significant effect was found in the behavioral study^14^, were presented as catch trials to avoid that participants provided automatic and stereotyped responses to the Morphed face. Each presented category consisted of 10 images at steps of 1% of morphing.

After each 2-minute synchronous/asynchronous IMS stroking block, participants evaluated 30 images from the Self, Other, and Morphed face categories (for a total of 90 images) plus 10 images from each of the Morphed-Self and Morphed-Other categories (catch trials) for a total of 110 faces per block, presented in random order (see Fig. 1A). Each self-other face discrimination trial (Fig. 1C) started with a fixation cross for 500 ms, followed by a face over a grey background (1000 ms), then by a blank gray screen (750 ms). Then, another fixation cross appeared for 250 ms, followed by 250 ms of blank gray screen, which signaled that a 20 cm vertical line (the extremities of which were labeled with 0 and 100) would appear next to let participants rate the extent to which each face represented themselves or the other. Each face image subtended about 6.54° × 8.98° degrees of visual angle, and was positioned about 70 cm from the observing subject.

Given that long-latency components may be influenced by task-relevance, to ensure that any illusory effect was not influenced by the instructions to rate the percentage of self or other, half of the participants reported the quantity of Self (Evaluate Self group; “How much does the image represent yourself?’ 0 = Other, 100 = Self) and the other half (Evaluate Other group) the quantity of Other facial features (‘How much does the image represent the other person?’ 0 = Self, 100 = Other) contained in any given face picture, as in our previous study^14^. Participants responded by moving a cursor along the VAS line and clicking the mouse at the estimated position. The position of the pixel marked on the screen was converted into a numerical value through an automated procedure.

### EEG recordings

EEG was continuously and simultaneously recorded from both pair participants using two BrainVision 64-channel systems. Electrodes were positioned according to the 10–10 international system. Electrodes’ impedance was checked in the beginning of the experimental session and kept below 5 kΩ throughout the experimental recordings. Electrodes were re-filled with conductive gel whenever necessary. The horizontal and vertical electrooculograms (EOG) were recorded at right external canthi and below the left eye, respectively. Given that also the EEG activity during the IMS stimulation was recorded, all scalp electrodes were referenced to the mastoid located ipsilaterally to the tactile stimulation side for each participant. Signals were filtered at 0.1 Hz, digitized at 2500 Hz, and stored on disk for off-line analysis.

### Subjective phenomenological experience of the illusion

Subjective reports about the perceived phenomenology of the illusion were collected after the synchronous and asynchronous IMS runs by asking participants to fill out a questionnaire used in our previous studies^14,18,19^ and adapted from the first study on the rubber hand illusion^69^. The questionnaire consists of three items designed to capture the experience of the illusion in its two components of referred sensation (statements 1 and 2) and sense of facial ownership (statement 3) and of 5 control items (statements 4–8). The control items are designed to describe experiences similar to those occurring during the enfacement illusion (but not evoked by it) and to control for the presence of a positive bias toward the experimenter’s expectations.

Participants indicated their response on a visual-analogue scale (VAS) that displayed ticks indicating agreement levels ranging from −3 (completely false) to +3 (completely true) and were instructed they could cross the line along its whole extension (thus also at intermediate positions between ticks).

Items list is presented in Table 1

**Table 1:** Table 1 reports the list of items assessing the perceived phenomenology of the illusion in its two components of referred sensation (statements 1 and 2) and sense of facial ownership (statement 3), and four control items (statements 5–8).

Questionnaire items
*STATEMENT 1****:**** It seemed as if I was feeling the touch of the stick in the location where I saw the other’s face touched*.
*STATEMENT 2: It seemed as though the touch I felt was caused by the stick touching the other’s face*.
*STATEMENT 3: I felt as if the other’s face was my face in terms of shape, skin tone, or other visual features*.
*STATEMENT 4:* It felt as if my face were drifting towards the other’s face.
*STATEMENT 5:* It seemed as if I might have more than one face.
*STATEMENT 6:* It seemed as if the touch I was feeling came from somewhere between my own face and the other’s face,
*STATEMENT 7:* It appeared as if the other’s face were drifting towards my own face.
*STATEMENT 8:* The other’s face began to resemble my own face

### Data Analysis

#### Self-Other Discrimination Task

Subjective self-other ratings underwent a 5 × 2 × 2 mixed model ANOVA with Identity [Self (~95% of Self); Morphed (~45% of Self); Morphed-Self (~55% of Self); Morphed-Other (~35% of Self); Other (~5% of Self)] and IMS Stroking (Synchronous; Asynchronous) as repeated measures factors and Instruction (‘Evaluate Self ‘; ‘Evaluate Other’) as between-subject factor. To analyze data, the VAS scores of the ‘Evaluate Other’ group (with scale ranging from 0 = self to 100 = other) were converted to the scale adopted by the ‘Evaluate Self’ group (where 0 = other and 100 = self).

Post hoc Newman-Keuls comparisons were used to test significant main or interaction effects.

#### Subjective phenomenological experience

A repeated measure ANOVA with factors Item (8 levels) and IMS Stroking type (2 levels) was run. Post hoc Newman-Keuls comparisons were used to test significant main or interaction effects.

#### ERPs analysis

EEG was re-referenced offline relative to the averaged mastoids, band-pass filtered (0.5–30 Hz) and segmented into 1100 ms epochs (−100 + 1000 ms) with respect to the appearance of the faces. The recordings were corrected automatically for eye movements and blinks according to the algorithm of^70^ (Brain Vision Analyzer, Brain Products GmbH, Munich, Germany). Semiautomatic artifact rejection was performed in order to discard epochs containing activity >65 µV. On average less than ~20% of the trials were rejected due to the presence of artifacts. One participant was excluded from further analyses due to excessive artifacts in the EEG signal. Baseline was calculated from 100 to 0 ms before the visual stimulus onset. Artifact-free trials were averaged separately for each participant and condition. Grand averages were then obtained for the Self-, Other-, Morphed- faces in both Asynchronous and Synchronous conditions (see Supplementary Fig. 1). Visual inspection of waveforms revealed the typical ERPs evoked by the visual presentation of faces (C1, P1, N170/VPP, P2 and LPP; please see Supplementary Fig. 1). Considering we used as online reference the mastoid ipsilateral to tactile stimulation side, the N170 component, which peaks over lateral temporo-parietal electrodes, was less pronounced than usual. Nevertheless, we clearly recorded a strong vertex positive potential (VPP), which is considered the functional counterpart of the N170^71^.

Even though we were specifically interested in testing the effect of IMS on self-face processing components occurring in the late temporal window where self-identification seems to occur, we wanted to exclude that any further IMS-related effect occurred in earlier time windows where self-specific, familiarity, as well as illusory facial agency^22^ effects were found.

Thus, for each condition the mean activity of each component was measured within a specific time window (as shown in Supplementary Fig. 1) centered on its peak and covering its ascending and descending phase (C1: 65–85 ms; P1: 90–115 ms; VPP: 135–180 ms; P2: 190–220. The late positive potential (LPP) was recorded from 300 ms up to the end of the segmentation and showed two peaks, one at about 390 ms and the other at about 625 ms. Thus two temporal windows were analyzed for early (350–415 ms) and late (450–750 ms) LPP. For each component, the electrodes where amplitude was maximal, as observed in grand-average waveforms, were grouped as follows: C1 (CP1-CPz-CP2- P1-Pz-P2); P1 (PO7-O1-POz-Oz-PO8-O2) according to side (left, middle, right); VPP (F1-FZ-F2- FC1-FCZ-FC2-C1-CZ-C2); P2 (POZ-PO1-PO2-PO3-PO4); LPP (FC1-FCZ-FC2-C1-CZ-C2-CP1-CPZ-CP2-P1-PZ-P2). To assess if the type of IMS stroking affected Self, Other and Morphed face visual processing, mean activity for each component underwent a repeated measure ANOVA with factors IMS Stroking type (Synchronous; Asynchronous), Identity (Self-; Other-; Morphed- Face), and Instruction (Evaluate Self; Evaluate Other). The P1 component showed a bilateral distribution and for this reason the factor Side (Right; Middle; Left) was added to the statistical model.

The Greenhouse-Geisser correction was applied to the *p*-values if sphericity could not be assumed. Post hoc Newman-Keuls comparisons were used to test significant main or interaction effects.

Data are available as Supplementary Information file.

## Results

### Self-Other discrimination after synchronous and asynchronous stroking

Statistical results substantially confirmed the enfacement effect previously found in a different sample^14^ and with a slightly different experimental design.

**Figure 2:**
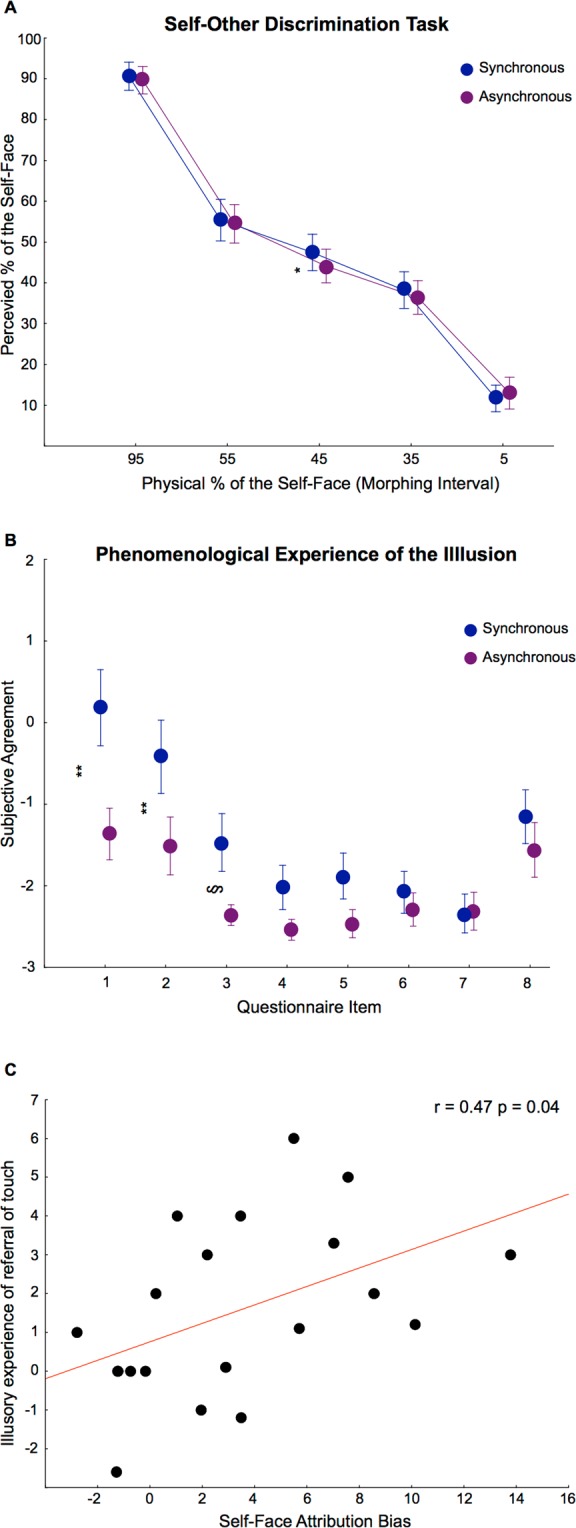
Behavioral correlates of the Enfacement illusion. (**A**) Implicit measure of the Enfacement Illusion: behavioral self-other discrimination task. Perceived amount of self-facial features (y axis) contained in the five clusters of face morphing (x axis). Remarkably, in the synchronous (blue line) with respect to asynchronous (violet line) condition participants rated as more similar to self, morphed faces containing more other than self-facial features (Morphed face cluster: 55% of Other- 45% of Self). *Asterisk indicates P < 0.003. (**B**) Explicit measure of the Enfacement Illusion: questionnaire items. The questionnaire (adapted from Botvinick & Cohen, 1998) included the eight statements reported in the Method section. Participants judged each statement on a seven-step visual-analogue scale ranging from −3 (‘completely false’) to +3 (‘completely true’). Synchronous (blue line) with respect to asynchronous (violet line) IMS produced more agreement on the three critical items describing the phenomenological experience of the illusion, both in terms of referred sensation (item 1–2) and, although marginally, also in terms of other’s face ownership (**for P = 0.000; § for P = 0.059). Points indicate mean responses. Bars indicate standard errors. (**C**) Correlation between the implicit and explicit measures of the Enfacement illusion. The graph displays the significant correlation (r = 0.47; p = 0.04) between the degree of referred sensations (item 1) (calculated as the difference between synchronous and asynchronous condition scores) and an index of the self-face attribution bias (calculated as the difference between synchronous and asynchronous subjective ratings of the critical interval of morphing (55% of Other- 45% of Self face).

No significant main effect or interaction of the factor Group was found (all F values < 0.77 and associated p > 0.47). There was no significant main effect of IMS (F_1,18_ = 3.034, p = 0.099). Importantly, a significant main effect of Identity was found (F_4,72_ = 316.44, η^2^ = 0.946, p < 0.001), showing that participants were able to perceive differently the five face categories (all of them were significantly different from each other; all ps < 0.0004). Even more importantly, a marginally significant interaction between type of IMS stroking and percentage of self-other morphing (Identity) was observed (F_4,72_ = 2.475, η^2^ = 0.121, p = 0.052) (Fig. 2A). Post hoc Newman-Keuls tests confirmed the presence of the enfacement effect: the Morphed faces (55% other −45% self on average) were rated as containing a higher percentage of Self, following the synchronous [(mean ± s.e.m), 47.43% ± 2.11; p < 0.003] with respect to the asynchronous IMS condition (44.13% ± 1.97).

### Subjective phenomenological experience

The 2 × 8 mixed-model ANOVA (with factors IMS Stroking and Item) showed a significant main effect of Item [F_7,133_ = 11.689, η^2^ = 0.381, p < 0.001], a significant main effect of IMS Stroking type [F_1,19_ = 10.181, η^2^ = 0.349, p = 0.005]. Crucially, the significant interaction of the two factors [F_7,133_ = 3.119, η^2^ = 0.141, p = 0.004; Fig. 2B] confirmed that synchronous stroking elicited higher agreement on the three critical items describing the phenomenological experience of the illusion, both in terms of referred sensation (item 1–2) [Item1: Synch (0.18 ± 0.46) (mean ± s.e.m) vs. Asynch (−1.36 ± 0.32), p = 0.000; Item 2: Synch (−0.42 ± 0.44) vs. Asynch (−1.51 ± 0.34), p = 0.001] and, although marginally, also in terms of other’s face ownership [Item 3: Synch (−1.47 ± 0.35) vs. Asynch (−2.36 ± 0.13), p = 0.059].

### Correlation between the self-bias and the phenomenological experience of the illusion

To understand whether the self-bias observed in the self-other discrimination task was linked to the phenomenological aspects of the illusion, we correlated the scores obtained in the first three items of the questionnaire with an index of the self-face attribution bias [i.e., self-attribution scores of the Morphed face (~45% of Self) after the synchronous minus the asynchronous IMS condition]. A significant positive correlation was found only for the first item (r = 0.47; p = 0.036, see Fig. 2C). Thus, the more the participants reported to feel like their own tactile sensation was originating from the face of the other person, the more they tended to incorporate features of the other’s face in the representation of their own face.

### Electrophysiological data

#### Synchronous IMS does not affect early stages of facial visual processing

Repeated measure ANOVAs with factors IMS Stroking, Identity and Instruction revealed no significant main or interaction effects for the C1 (all F < 2.581; all p > 0.13), the VPP (all F < 2.329; all p > 0.11) and the P2 (all F < 3.716; all p > 0.07) components. Repeated measure ANOVA with factors IMS Stroking; Identity; Instruction and Side (left; middle; right) was run for the P1 component. We found only a significant main effect of Side [F_2,34_ = 8.183, η^2^ = 0.325, p = 0.001], with higher amplitudes recorded on right (3.28 ± 0.81) and left (2.75 ± 0.67) compared to midline (1.57 ± 0.81) electrodes (all ps < 0.01). No other significant main or interaction effects were found (all F < 2.771; all ps > 0.11).

#### Synchronous IMS affects late stages of self-face visual processing

Visual processing of the three faces differed only at longer latency ranges, where an initial effect of face familiarity (i.e., the self and friend faces differed from the morphed face in the early LPP range) preceded the ability to discriminate the three face identities in the late LPP range.

Repeated measure ANOVA on the early LPP (350–415 ms) with factors Stroking, Identity, and Instruction, showed only a main effect of Identity (F_2,34_ = 26.083, η^2^ = 0.605, p < 0.001). Newman-Keuls post-hoc revealed that at this processing stage the brain discriminated between exposed and unexposed faces, with the Self (7.13 µV ± 0.66) and Other (7.02 µV ± 0.59) faces evoking higher amplitudes than the Morphed face [(5.53 µV ± 0.61), all ps < 0.001], which is a type stimulus that participants have never seen before.

**Figure 3:**
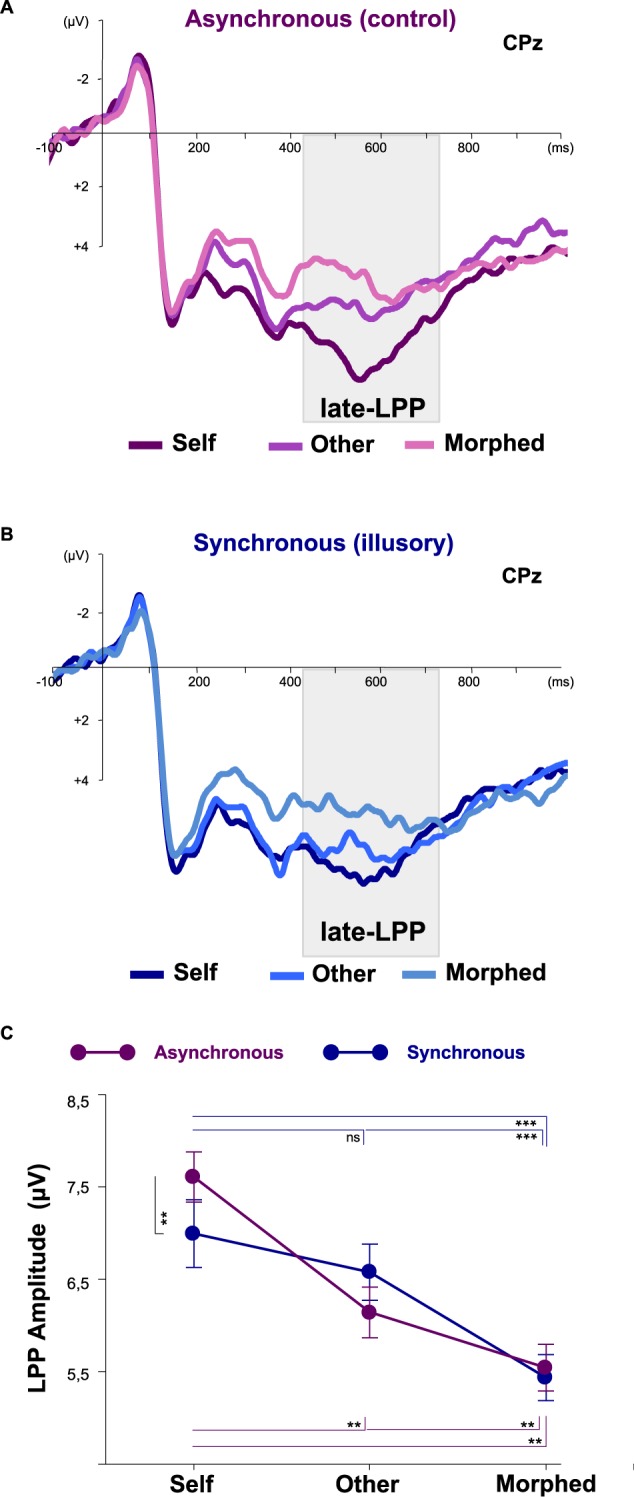
Electrophysiological correlates of the Enfacement illusion. Waveforms recorded from CPz electrode and topographical distribution on the scalp of the Late Positive Potential (450–750 ms) elicited by the presentation of the Self-, Other- and Morphed (45% Self-55% Other) faces after (**A**) Asynchronous (graduated violet lines) and (**B**) Synchronous (graduated blue lines) IMS stroking are shown. (**C**) A significant double interaction between IMS Stroking and Identity was found showing that in the asynchronous condition the Self evoked higher amplitudes with respect to the Other, and both Self and Other faces evoked higher amplitudes than the Morphed face. Illusory self-other merging, instead, reduced amplitudes evoked by the Self face with respect to the asynchronous condition, and made the Self-face processing become similar to the processing of the Other-face. No differences emerged in the amplitudes of the Morphed and Other faces between the illusory and control condition. Bars indicate standard errors. Asterisks indicate significant differences between conditions (***P < 0.001; **P < 0.029).

Interestingly, synchronous visuo-tactile stroking affected the way that Self-, but not Other- and Morphed-, faces are processed in the following time window that appeared sensitive to the self-face recognition stage^30,44,45,47,48,54^. Repeated measure ANOVA on the late LPP (450–750 ms) showed a significant main effect of Identity [F_2,34_ = 16.005, η^2^ = 0.485, p < 0.001], indicating that the three identities are discriminated at this stage with Self faces (7.27 µV ± 0.46) producing higher amplitudes than Other Faces (6.35 µV ± 0.48) and both were significantly higher than Morphed Faces [(5.47 µV ± 0.49); all ps < 0.01)]. Further, a significant interaction between Identity and IMS Stroking was found (F_2,34_ = 4.290, η^2^ = 0.201, p = 0.022), showing that visual processing of the three identities changed as a function of the type of IMS stroking received. In the asynchronous stroking condition (Fig. 3A,C and Supplementary Fig. S2) the response pattern of the three faces categories remained unchanged with respect to the main effect of Identity [Self (7.58 µV ± 0.49) > Other (6.12 µV ± 0.50) and Morphed (5.53 µV ± 0.52), Other > Morphed, all ps < 0.029]. In the synchronous IMS (Fig. 3B,C and Supplementary Fig. S2) the late LPP elicited by the Self-face (6.96 µV ± 0.54) was reduced (p = 0.026) compared to the asynchronous stroking condition; however, Morphed and Other face processing did not differ across stroking conditions (Synchronous-Morphed vs. Asynchronous-Morphed, Synchronous-Other vs. Asynchronous-Other; all ps > 0.11). Finally, while experiencing the enfacement illusion, the Self-face processing was no more different from the Other-face processing [Synchronous-Self vs. Synchronous-Other (6.59 µV ± 0.55), P = 0.12], while both differed from the Morphed face [Synchronous-Self vs. Synchronous-Morphed (5.42 ± 0.50), and Synchronous-Other vs. Synchronous-Morphed, all ps < 0.001].

## Discussion

In this study, we report EEG correlates of the enfacement illusion and thus provide a direct evidence that synchronous IMS causes a change in the neural processing associated to self-face representation.

In keeping with studies performed in our own^14,18,19^ and other laboratories^12,13,15–17,72^, the present behavioral results show that participants experience the enfacement after synchronous but not after asynchronous IMS. Tellingly, enfacement was experienced at both implicit (i.e., self-face attribution bias of morphed faces containing more other- than self- facial features) and explicit (i.e., the items describing the phenomenal experience of the illusion) levels. We also found a significant correlation between the subjective experience of referred sensation and the self-face attribution bias during the enfacement illusion suggesting a clear link between visuo-tactile integration processes and changes of the sense of facial identity.

Recording ERPs elicited by the Self-, Other- and Morphed- faces after participants received synchronous and asynchronous IMS stimulation allowed us to explore with high temporal resolution all the stages of visual processing at which enfacement-related modulation could occur. Since multisensory integration is called into play while participants receive IMS stimulation and we recorded ERPs from the faces only after IMS ended, what we observe in ERPs is the posthumous effect of multisensory integration over the different stages of face processing.

A completely novel finding of the present study is that such modulation specifically occurs at late stages of visual self-face processing. Differently from Serino *et al*.^22^, we found that early featural, configural and fine-grained perceptual analyses of faces (as indexed by the C1, P1, N170/VPP and P2 components, respectively^26,27,71^) were not affected by either IMS or by the identity of presented faces. Several differences between the present experimental paradigm and the one used by Serino and colleagues’ (2015) may have contributed to these discrepant results. The first is that we tested pairs of participants well known to each other. This may have reduced the possible confound of higher familiarity of the self-face with respect to the other face, which may result in early ERPs differences between the self and the control face^47^. Serino and colleagues (2015), instead, compared the self-face with a previously unknown and unfamiliar avatar’s face. The second and probably most important difference may reside in the presentation times of the facial stimuli (200 ms^22^ vs. 1000 ms in our study) and the type of requested responses (i.e., an immediate and time-constrained two alternative forced choice^22^ vs. a delayed and time-unconstrained response concerning the amount of self and other facial features in our study). Different presentation times and response types may have called into play different perceptual and cognitive processes necessary to accomplish the task. For example, in the case of a time-constrained and forced-choice response, the judgment could be entirely based on a quick and rough estimation of how much the overall configuration of the observed face matches the self-face representation. The only reference point could be the self-face representation, since the other face was unfamiliar. In contrast, when a time unconstrained response was allowed and a precise estimation of the amount of self and other facial features is requested, the stimulus judgment could rely on the accurate computation of how much the single facial features (e.g., their form, dimension, color) and their overall spatial configuration differ from the internal self-face representation. Also, since the other face belonged to a personally known participant, a representation of his/her face is available in memory and may contribute to the final response. Thus, future studies are necessary to provide a definitive answer to the issue of how IMS may change early perceptual (vs. late) processing of faces.

Importantly, we found that face identity influenced early-LPP amplitudes in a late time window (350–415 ms) similar to the one (300–550 ms) where previous studies found that familiar, emotionally and personally relevant faces are distinguished from other faces^29,30,32,73^ [but see also^28,42^ for earlier effects of familiarity]. In keeping, the self and friend faces produced larger ERPs amplitude as compared to morphed faces. This effect might be explained by the fact that morphed faces – despite being a combination of the well-known self and friend faces- are still unfamiliar in the sense that they have not been seen before and cannot be associated with any real unique and distinct identity, emotional, or semantic information.

Crucially, in accordance with previous literature^44–47^ we found that in a later time window (450–750 ms), the LPP amplitudes discriminated between Self-, Other- and Morphed- faces. More specifically, and as predicted, highest amplitudes were found for Self faces with progressively lower amplitudes for progressively less familiar Other and Morphed face.

The most relevant result, however, was the significant interaction between Identity and IMS-Stroking type. Indeed, while the above-mentioned pattern of amplitudes elicited by the three identities was present after asynchronous stimulation, experiencing enfacement selectively changed the way the self-face was processed. Late-LPP amplitudes produced by the Self-face were reduced, while Morphed- and Other- face late-LPP amplitudes did not change after synchronous- vs. asynchronous-IMS. Also, experiencing the enfacement illusion cancelled out the difference in late-LPP amplitude between Self-and Other-face in the asynchronous control condition. In keeping with our predictions, the late-LPP effect occurred at a processing stage that previous literature linked to self-processing, motivated attention toward emotionally salient stimuli^37^, memory retrieval of self-face specific information^30,44–46,48,50,53,54,62^, and particularly to the identification of the current from the past self-face^47^.

As previously mentioned, the visual representation of one’s own face is built and continuously updated on the basis of the congruent multisensory signals one experiences when looking at oneself in the mirror. This is reminiscent of what happens in synchronous IMS where the felt touch is surprisingly mirrored on what seen on another’s face. However, since participants cannot move (and thus check whether the observed face is their own), the brain may attempt to minimize surprise by including facial features of others into self-face representation^20^. Our results fit well with this hypothesis by showing that enfacement selectively affects neural visual processing of the self-face and add to previous studies on the neural correlates of enfacement, in which brain activity was recorded during interpersonal multisensory faces’ stimulation^21,22^. Apps and colleagues (2015) found that activity in unimodal (inferior occipital gyrus) and multimodal [inferior parietal sulcus (IPS) and temporo-parietal junction (TPJ)] cortices was modulated during spatially in/congruent and temporally a/synchronous visuo-tactile stimulations and varied parametrically with the illusory experience of the ‘looking at my face’ reported at the end of the stimulation phase. Based on these and previous results on IMS-induced bodily illusions, we proposed a tentative neural account of enfacement^11,74^, hypothesizing that TPJ would detect and inform IPS of a conflict between tactile afferent and visual signals that, although congruent with self-percept, originate instead from another person’s face. Indeed, thanks to its anatomical and functional properties IPS is crucial for maintaining a coherent body representation during multisensory conflicts^75,76^. For example, IPS is involved in recalibrating the peri-hand space representation toward that of the synchronously stimulated rubber hand [so that tactile, visual, and proprioceptive signals fuse in a single coherent percept^77^] in the temporal window that precedes the illusory sense of ownership^75^. Accordingly, during synchronous facial IMS, ventral IPS could remap the space around the face as seen in a mirror and thus suppress the conflict between tactile stimuli applied to the self and temporo-spatial congruent visual stimuli applied to another face. This process would result in updating the self-face representation to include facial features of the synchronously stimulated other and thus in prompting the illusory perceptual experience of looking at oneself in the mirror.

Here we highlight a possible electrophysiological correlate of this ‘including-the-other-into-the-self’ process by showing reduced self-face late-LPP amplitudes and similar processing of the self- and other-face after synchronous IMS. Although speculatively, we submit that synchronous IMS induces a newly formed self-face representation which, being the result of a temporally limited manipulation, should be quite unstable. Reasonably, there should persist a memory trace of the habitual self-face representation consolidated through daily life experience with the mirror. We think that our late-LPP effect indexes changes of self-face representation derived from a comparison of the observed usual self-face (i.e., the 100% self-face presented on the screen) with the newly induced self-face representation. Consequently, the self-face observed on the screen does not match the online current representation of the self, resulting in decreased late-LPP amplitudes and in neural processing similar to the one occurring for the familiar other’s face. Highest self-face late-LPP amplitude after asynchronous stimulation may index instead that online current self-representations match the observed ones. This interpretation explains also why we found that the synchronous IMS affected the 100% self-face visual processing but not the behavioral responses to the 100% self-faces. Indeed, the subjects may still rely on the robustly stored visual self-representation upon explicit judgments, while the ERPs implicit measure of the visual self-face processing indicates that the presented “usual” self-face does not match with the newly induced self-face representation. On the contrary, the morphed face (55% other- 45% self) may match more with the newly updated mental self-face representation. Given this match, participants may judge morphed images as containing more self with respect to the asynchronous stimulation (where no conflict between stored and newly updated self-face representation occurs). Indeed, the asynchronous condition shows the same ERPs identity-related amplitude pattern found in previous self-other face recognition studies where no IMS was applied, and unaltered discrimination of self-other facial features in the morphed images where self-attribution scores did not differ between asynchronous and baseline conditions^14^.

Studies suggest that LPP amplitude is modulated by personally salient, rewarding and emotional stimuli as well as by motivated attention^34,61,78,79^. Thus, late-LPP amplitudes evoked by the self-face after synchronous-IMS might also reflect enfacement-related reduction of self-face emotional salience. In particular, synchronous IMS may reduce the emotional salience of self-representation features and make it similar to that of other face. However, we posit that our LPP modulation reflects the process of ‘including the other into the self-face’ (and in particular the matching of the observed self-face with the newly IMS-induced self-face representation). Indeed, our result is in keeping with a study^47^ where late latency range components discriminated actual self-face from past self-face and co-twin face, possibly because of different mnemonic retrieval of self-specific information. Moreover, the reduction of the emotional salience of self-face stimuli can occur only after changes to the self-representation have occurred. At any rate, future studies should test the intriguing hypothesis that enfacement modifies the unique emotional content of the self-face.

To conclude, we would like to consider some limitations and future developments of the present study. First, we want to point out that our behavioral results replicate previous studies and thus can be considered robust and reproducible. However, our result on the IMS-induced modulations of self-face neural processing is a novel one and has to be explored further in future studies. Indeed, due to the complexity of the present experimental design, we tested a number of participants that, although higher than the one used in the only previous ERP study on this topic^22^ and well in the range of standard ERP studies, may be considered limited. Similarly, the lack of IMS-induced modulation of early perceptual processing of faces should be investigated further with ad-hoc experimental designs adept to take into account the interindividual variability in perceiving morphed faces as belonging to the self or to the enfaced other. Another important issue to investigate in further ad-hoc studies regards how inter-individual differences in the degree to which the self- and other representations overlap at baseline (i.e., before and/or independently of any IMS manipulation) may influence neural and behavioral measures of enfacement. Indeed, previous studies showed that distinctiveness of self-representations, as indexed by how rapidly neurotypical participants switch their judgments from self to other in a self-other recognition task (i.e., the steepness of the sigmoidal curve around the midpoint), is influenced by autistic traits^80^ and may influence self-other face perception^81^. Thus, an interesting hypothesis to test is whether individuals with a more distinct self-face representation at baseline (i.e. higher slope for the self-other psychometric function) show less IMS-induced malleability of self-face representation, compared to individuals who have more overlapping representations of self and other faces (i.e. low slope values for the psychometric function).

## Conclusions

Overall, the present study provides direct evidence that enfacement may powerfully induce plastic changes of the self-face representation and that features of others’ identity can be included in the notion of self. It’s worth noting that the plasticity of the self-representation (i.e., as indexed by magnitude of the enfacement effect) seems to be highly dependent upon positive interpersonal perception of the synchronously stimulated other (i.e., in terms of physical attractiveness or personality traits^14,18^). Thus, research on enfacement will be of potentially great importance for understanding deficits of self-representation and self-other interactions.

## Supplementary information

Bufalari_Supplementary Figure 1 and 2
