# Supplementary material for: Malleability of the self: electrophysiological correlates of the enfacement illusion
Source: Sci Rep. 2019 Feb 8;9:1682. doi: 10.1038/s41598-018-38213-y (PMC6368628; doi:10.1038/s41598-018-38213-y)

Malleability of the self: electrophysiological correlates of the enfacement illusion

SHORT TITLE: Enfacement changes self-face processing

Ilaria Bufalari[1,2,*], Anna Laura Sforza[2,4], Francesco Di Russo[2,3], Lucia Mannetti[1], Salvatore Maria Aglioti[2,4]

Dipartimento dei Processi di Sviluppo e Socializzazione, Università degli studi di Roma "La Sapienza", Rome, Italy
IRCCS Fondazione Santa Lucia, Rome, Italy.
Dipartimento di Scienze Motorie, Umane e della Salute, Università degli Studi di Roma "Foro Italico", Rome, Italy
Dipartimento di Psicologia, Università degli studi di Roma "La Sapienza", Rome, Italy.

Corresponding Author:
* Ilaria Bufalari
E-mail: ilaria.bufalari@uniroma1.it (IB)

Supplementary Figure 1. Waveforms (A) and topographical distribution on the scalp (B) of Event Related Potentials (ERPs) evoked by the visual presentation of the faces. Grand-averages are shown for FCz, CPz, POz and PO8 (right panel) electrodes where the different ERPs components were recorded with maximal amplitude. Mean activity of each component was measured within a specific time window (grey rectangles) centered on the component's peak.

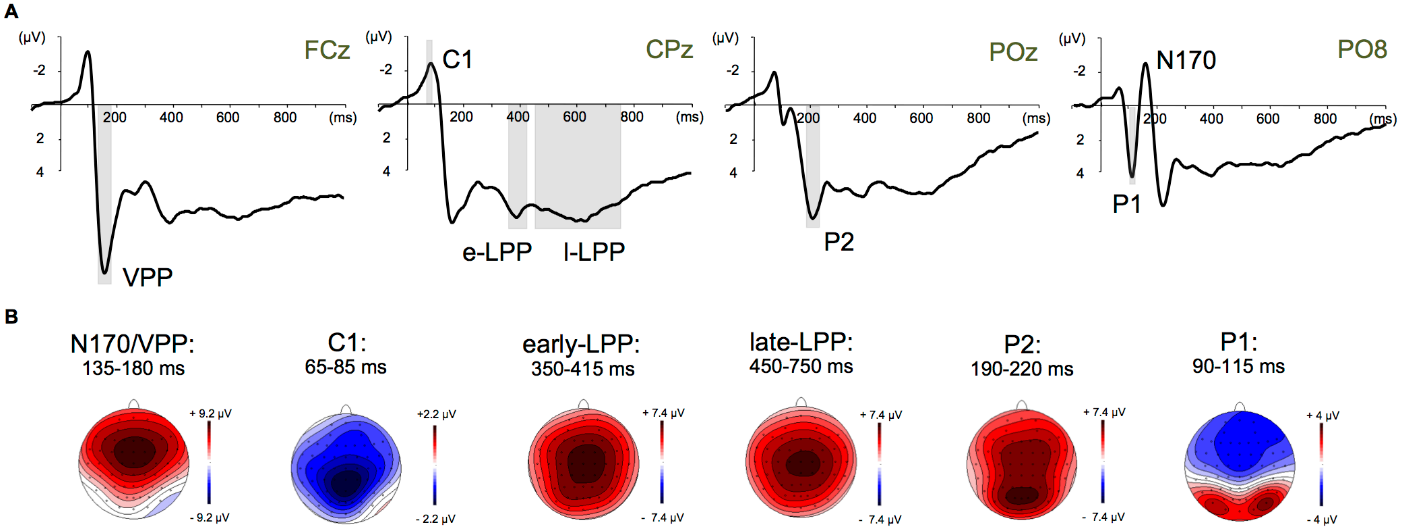

Supplementary Figure 2. Waveforms representing the Event Related Potentials (ERPs) evoked by the visual presentation of the faces in the Synchronous (A) and Asynchronous (B) conditions. Grand-averages are shown for FCz, CPz, POz and PO8 electrodes where the different ERPs components were recorded with maximal amplitude.

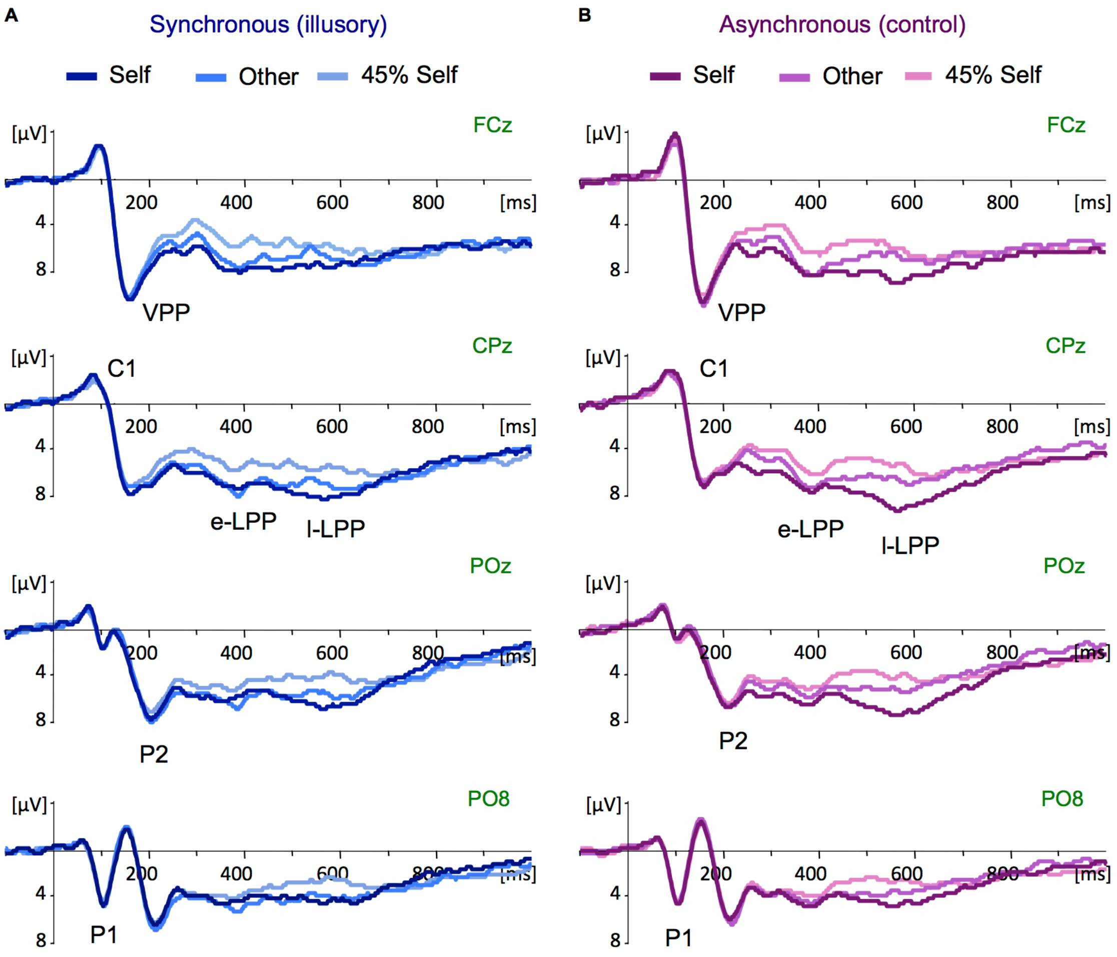

Supplement: Supplementary file 1 — Bufalari_Supplementary Figure 1 and 2 [file 41598_2018_38213_MOESM1_ESM.pdf]
